# Classical Food Quality Attributes and the Metabolic Profile of Cambuci, a Native Brazilian Atlantic Rainforest Fruit

**DOI:** 10.3390/molecules26123613

**Published:** 2021-06-12

**Authors:** Poliana Cristina Spricigo, Banny Silva Barbosa Correia, Karla Rodrigues Borba, Isabela Barroso Taver, Guilherme de Oliveira Machado, Renan Ziemann Wilhelms, Luiz Henrique Keng Queiroz, Angelo Pedro Jacomino, Luiz Alberto Colnago

**Affiliations:** 1 Luiz de Queiroz College of Agriculture, University of São Paulo, 11 Pádua Dias Ave., Piracicaba 13418-900, São Paulo, Brazil; isabela.taver@usp.br; 2 Institute of Chemistry of Sao Carlos, University of São Paulo, 400 Trabalhador São Carlense Ave., São Carlos 13566-590, São Paulo, Brazil; banny.barbosa@gmail.com (B.S.B.C.); guilherme.oliveira.machado@usp.br (G.d.O.M.); 3 Embrapa Instrumentation, 1452 XV de Novembro Street, São Carlos 13560-970, São Paulo, Brazil; borbakr@gmail.com (K.R.B.); luiz.colnago@embrapa.br (L.A.C.); 4 Chemistry Institute, Federal University of Goiás, Esperança Ave., Goiânia 74690-900, Goiás, Brazil; renan.ziemann@gmail.com (R.Z.W.); keng@ufg.br (L.H.K.Q.J.)

**Keywords:** Brazilian fruit, metabolites, NMR, accession

## Abstract

The cambuci is a native Brazilian fruit from the Atlantic Forest biome. A soft and astringent pulp, a green color, and a sweet aroma are its main characteristics. Classical food quality attributes (fresh fruit mass, fruit height, diameters, total soluble solid, titratable acidity, and ratio) and the metabolic profile from ten accessions from three different locations were analyzed herein by analytical methods (refractometry and neutralization titration) and nuclear magnetic resonance spectroscopy. Concerning sugar content, sucrose was the predominant compound, with glucose and fructose alternating in second, depending on the accession. Citric acid was the most relevant acid, followed by shikimic and quinic acids in quite variable amounts. These three main acids vary in amounts for each accession. Ascorbic acid content emerges as an important quality attribute and makes this fruit nutritionally attractive, due to values comparable to those contained in citric fruits. The main amino acids identified in cambuci were glutamic acid individually or in comprising the tripeptide glutathione (glutamic acid, cysteine, glycine). The quality diversity of the evaluated accessions suggests the potentiality of cambuci use in future breeding programs.

## 1. Introduction

Cambuci (*Campomanesia phaea*) is a native Brazilian Atlantic Rainforest fruit. It is an endangered species that naturally occurs in a mountainous relief area, named Serra do Mar, mainly in the state of São Paulo and in smaller numbers in the states of Rio de Janeiro and Minas Gerais. Belonging to the *Myrtaceae* botanical family, cambuci contain a fleshy and soft pulp when ripe, a thin skin and persistent green coloration. All cambuci parts (peel, pulp and seed) are edible, resulting in high yields and the total use of this fruit. It also presents a sweet and attractive aroma with a peculiar astringent taste. At room temperature, cambuci postharvest longevity extends only for up to 3 to 4 days, which may limit its *in natura* use. In this regard, the fruits are often frozen, which allows them to be used in preparations for an extended period of time. Despite the viability of frozen cambuci, its current use is restricted to the local population, who consumes it prepared as juices, ice creams, and jellies. Nowadays, cambuci fruit does not have an international demand; however, its commercial products should be stimulated.

Cambuci exhibits great potential for human consumption given its sensory characteristics, while it is also of interest for industries focused on processed food, cosmetics and pharmaceutical products. Some assessments have listed a few quality attributes of this fruit, although a metabolic approach is still lacking [1,2,3]. The *Campomanesia* genus is recognized as being rich in phenolic compounds and very promising for nutraceutical and pharmaceutical applications. These compounds are related to antioxidant properties, acting as anti-inflammatory, anti-diabetic and antimicrobial compounds, as well as demonstrating protective cardiovascular system effects [4]. Scientific investigations concerning cambuci composition and uses contribute to at least two aspects of society’s progress: stimulating species and native environment preservation, and the availability of health-friendly food. 

Chemical fruit profiles are a combination of primary and secondary metabolites, which confer intrinsic quality characteristics to each species [5]. Metabolomics based on nuclear magnetic resonance (NMR) spectroscopy have been widely used to determine the chemical profile of fruits during their postharvest period and, therefore, assist in understanding their composition [6,7,8]. NMR sample preparation is relatively easy to perform and allows for qualitative and quantitative evaluations of a wide range of metabolites in complex mixtures, such as those observed in biological samples [5]. Thus, this technique is able to offer a clearer identification of fresh fruit profiles, resulting in less biochemical modifications due to manipulation. 

This paper presents, for the first time in the literature for cambucis, the metabolomic profile of the fruit analyzed by NMR with emphasis on sugars, acids, amino acids and total polyphenols. In this context, the postharvest food quality attributes and metabolic profile of cambucis from ten accessions from three different locations using both classical methods and NMR spectroscopy were investigated herein. 

## 2. Materials and Methods

### 2.1. Samples

Cambucis from ten accessions were obtained from three cities in the state of São Paulo, Paraibuna (accessions 01 and 02) (23° 23’ 09” S 45° 39’ 43” O), Mogi das Cruzes (accessions 03, 04, 05, 06 and 07) (23° 31’ 22” S 46° 11’ 16” O), and Salesópolis (accessions 08, 09 and 10) (23° 31’ 55” S 45° 50’ 45” O), located in a region known as Serra do Mar. Paraibuna and Salesópolis are neighboring municipalities, but climatically different according to the Köppen-Geiger classification. Paraibuna is located within the metropolitan region of São Paulo and is classified as a temperate oceanic climate (Cfb), while Salesópolis is located in Paraíba valley and the northern coast of the São Paulo region, presenting a humid subtropical climate (Cfa). Mogi das Cruzes is part of the metropolitan region of São Paulo and Alto Tietê and is inserted in a humid climate territory with monsoon influence (Cwa). The Cfa and Cfb climates are humid, while the Cwa presents a dry season during the winter. In the summer, Cfa and Cwa reaches temperatures of over 22 °C, in contrast with CFb, where this temperature is not reached. Therefore, despite the proximity of the fruit sampling areas, temperature may influence fruit quality. Typically, the cambuci harvest is carried out in the summer–autumn seasons in the southern hemisphere, from February to June.

Forty-four fruits collected from each accession were used in the present study, totaling four hundred and forty fruits. The samples were then transported to the Laboratory of Postharvest of the Horticultural Products, at the Luiz de Queiroz Superior School of Agriculture (ESALQ-USP) and to Embrapa Instrumentation, where they were frozen at −20 °C.

### 2.2. Physicochemical Analyses

Fresh fruit masses (g), were determined using an analytical balance. Fruit heights and diameters were measured using a digital caliper and expressed as millimeters (mm). Total soluble solid contents (SSCs) were determined by using method 932.12 [9], with a digital refractometer (Atago brand, Palette 101, Tokyo, Japan) expressed as °Brix. Titratable acidity (TA) was determined by neutralization titration, using a digital potentiometer (Tecnal, Tec 03 MP) and the results were expressed as % citric acid (method 942.15 [9]). SSC and TA values were used to calculate the SSC/TA ratio. Fresh mass, height and diameter were determined by the analysis of 30 fruits (three replicates containing ten fruits each). The soluble solid and total titratable acidity analyses were performed using portions of the same nine entire fruits (three replicates containing three fruits each).

### 2.3. Metabolite Extraction for NMR Analyses

Pulps of each fruit (~50 g) was individually crushed using a mixer and centrifuged (*Eppendorf MiniSpin 11 mm diameter*) at 14,000 rpm for 30 min. The supernatant juices were then separated from the fibers and kept at 4 °C (~25 mL). The 50 juice samples (quintuplicates of the ten accessions) were divided in two analytical replicates, yielding 100 NMR samples. The maximum quantification errors between the two NMR analyses were lower than 15%. A total of 450 µL of the supernatants were solubilized in 450 µL of deuterium oxide phosphate buffer (0.10 M, pD = 7.3) containing 0.050% w/w of sodium 3-trimethylsilyl-2,2,3,3-d4-propionate (TMSP-d4, from Cambridge Isotopes, Leicestershire, UK) and 0.02% m/v of sodium azide. The pH of each sample was adjusted to 3.00 ± 0.02 using microliter amounts of 1 M NaOH or 1 M HCl solutions. Subsequently, 550 µL were transferred to a 5 mm NMR tube. 

### 2.4. NMR Analyses 

NMR spectra were obtained at 298 K using a 14 T Bruker Avance III spectrometer (Bruker BioSpin, Rheinstetten, Germany), 5 mm PABBO probe head with gradients, automated tuning and matching accessory (ATMATM), BCU-I for temperature regulation and a Sample-Xpress sample changer. A protocol (90° calibrated pulse and irradiation at the water frequency) was first established for a QC sample (quality control a mixture of samples). Then, this protocol was run in fully automatic mode using Bruker routines (load, auto-tune, lock, phase, flicker, acquisition, process) for each sample. Spectra were acquired for proton NMR using the NOESY-presaturation pulse sequence (Bruker 1D noesygppr1d pulse sequence), 64 k data points, spectral width of 20.0276 ppm, acquisition time of 2.726 s, recycle delay 4s), dummy scans of 4, accumulation of 256 transients, mixing time of 0.005 s, and fixed receiver gain (45.2). FIDs were multiplied by a 0.3 Hz exponential multiplication function prior to the Fourier transformation. Phase and baseline corrections were carried out using the TopSpin^TM^ 3.6.1 software (Bruker, Biospin, Germany). The TMSP-d4 signal was calibrated at δ 0.00. The 1D spectra were assigned using the literature and databases values, in which overlap signals of each compounds were deconvoluted using the software Chenomix NMR Suite 8.4 (Chenomix Inc, Edmonton, Canada), then the identity of compound was confirmed using the 2D NMR experiments ^1^H JRES, ^1^H-^13^C HSQC, and ^1^H-^1^H COSY were performed on selected samples, by the correlated signals between vicinal hydrogens (COSY), the correlated signals between carbon and correspondent hydrogen (HSQC), and J-coupling constant between protons of a specific signal (JRES). Metabolite peaks were integrated and quantified relative to the “Electronic to Access In Vivo Concentration 2” (ERETIC2) signal, referenced using a 2 mM sucrose standard and the fixed RG used for the samples. It was used the absolute area of integrals, molar mass, and the number of hydrogens of the respective NMR signal of each compound in the identified peaks to calculate concentration in mM, and then their respective mg 100 g^−1^. 

### 2.5. Data Processing

The ^1^H-NMR spectra data, from 0 to 8.5 ppm, were aligned using the Icoshift algorithm [10], followed by optimized bucketing [11] (sample interval: 0.05 ppm, slackness: 50%) and transformation into a data matrix, using the MATLAB v. 9.10 (The MathWorks, Inc, Natick, MA, USA) [12] and R software packages [13]. Metabolite quantification was analyzed for variance and classification of means by Tukey’s test. For the pathway analysis, differential metabolites were cross-listed with the pathways available at the Kyoto Encyclopedia of Genes and Genomes (KEGG), using previous identifications for *Arabidopsis thaliana* (thale cress) (KEGG), PubChem Compound, Chemical Entities of Biological Interest (ChEBI), Japan Chemical Substance Dictionary Web (NIKKAJI), and Chemical Abstracts Service (CAS). The top altered pathways were identified and built according to the potential functional analysis, using the MetaboAnalyst 4.0 platform [14].

### 2.6. Statistical Analysis

Data were subjected to an analysis of variance and mean comparisons were performed using the Tukey test (*p* < 0.05). The coefficient of variation (CV) is the ratio of the standard deviation to the means.

Results of the quality parameters analyzed by the analytical method and by NMR were submitted together to principal component analysis and correlation using GraphPad Prism v.8 (GraphPad Software Inc, San Diego, CA, USA) and Statistica v.7 software (Statsoft Inc, Tulsa, OK, USA).

## 3. Results and Discussion 

### 3.1. Physicochemical Parameters

The physicochemical parameters of the investigated cambucis reflected accession diversity, as expected, since cambuci have not yet been selected in genetic breeding programs (Table 1). The average fresh fruit mass was 57.0 g (CV: 26.2%). Small producers and fruit collectors from the Serra do Mar region employ an informal cambuci classification, where large fruits are considered as those over 68 g, medium, from 39 g to 68 g, and small, less than 39 g [3]. In this study, fresh masses ranged from 36.3 to 88.3 g. Fruit height (h) was always smaller than their diameter (d), with and h/d ratio <1, indicating flat fruits in accordance with [3,15].

The average SSC of cambuci was 10.8 Brix, ranging from 7.8 to 14.0 Brix. These values are similar to the SSC observed in other chemical characterization studies in cambuci, which reported values between 7.3 to 13.3 Brix and 5.1 to 11.0 Brix [3,16]. TA values ranged from 1.1% to 4.3%, which is a large variation from that observed previously—1.3% to 2.9% (citric acid eq.) [3].

For comparison, in a study evaluating the quality of passion fruits, 9.1%, 11.6% and 12.3% of soluble solids were reported for yellow, purple and orange fruits, respectively [17]. Similarly, these same fruits contained total titratable acidity values of 2.1%, 2.8% and 2.2%. Less acidic cambucis exhibited TA similar to the average observed in oranges, of 1.0%, while high acidity cambucis are similar to acid limes, presenting TA between 5–6% [18].

When the SSC/TA ratio is considered as a quality attribute, each fruit will present its own scale, based on sugar and acid contents, as well as other minority components. The SSC/TA ratio can be used as a discriminating quality criterion in fruit, as described previously for peaches and nectarines [19]. When 39 peach and nectarine cultivars were evaluated, the variation ranged from 9.36 to 92.8 [19]. For the cambucis assessed in the present study, the variation ranged between 3.1 and 8.0.

Usually, cambuci fruits present the following values of other physical and chemical properties: moisture content 86–90%, pH 2–3, fibers 0.1–0.2%, total lipids 0.02–0.05%, proteins 0.02–0.03%, carbohydrate 0.9 g Kg-1, ash content 0.02%, and mineral profile comprising nitrogen 0.01–0.03, phosphorus 0.01, potassium 0.1, calcium 0.001–0.005, magnesium 0.01, sulfur 0.01, boron 0.1, zinc 0.1, iron 0.2–0.3, manganese 0.02–0.1, copper 0.02–0.04, sodium 0.3–0.6 g kg^−1^ DW [3,5,20]. These parameters were previously reported presenting genetic variability values for fruit characters once, when different accessions were evaluated, the coefficient of variance was demonstrated as high levels (>20%), as Bianchini et al. show [21]. However, there is no report of the complete metabolite profile as a parameter of chemical changes to the point the variability genetic of cambuci fruits as describe in the next section. 

### 3.2. NMR Spectra Metabolite Assignment

**Figure 1:**
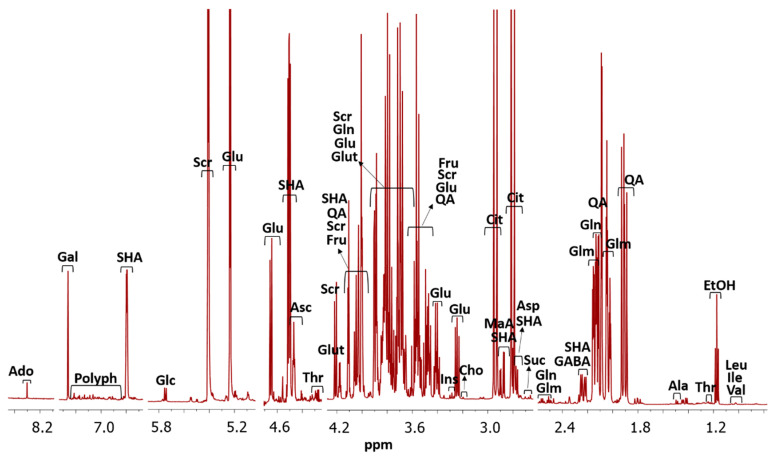
Typical ^1^H NMR spectrum of the cambuci supernatant. The assigned metabolites are as follows: Ado- adenosine-like substances, Ala: L-alanine, Asc: ascorbic acid, Asp: L-aspartic acid, Cho: choline, Cit: citric acid, Eth: ethanol, Fru: fructose, GABA: gamma-aminobutyric acid, Gal: gallic acid, Glc: glucose 6 phosphate, Glm: glutamic acid, Gln: L-glutamine, Glu: D-glucose, Glut: glutathione, Ins: inositol, Ile: L-isoleucine, Leu: L-leucine, MaA: malic acid, Polyph: polyphenols, QA: quinic acid, Scr: sucrose, SHA: shikimic acid, Suc: succinic acid, Thr: L-threonine, Val: L-valine.

A typical cambuci NMR spectrum of 0.9–8.5 ppm is presented in Figure 1. Twenty-five metabolites (adenosine-like substances, alanine, ascorbic acid, aspartic acid, choline, citric acid, ethanol, fructose, gamma-aminobutyric acid, gallic acid, glucose-6-phosphate, glutamic acid, glutamine, glucose, glutathione, inositol, isoleucine, leucine, malic acid, quinic acid, sucrose, shikimic acid, succinic acid, threonine and valine) were assigned. This number of compounds, as well as the compounds identified, are in agreement with the literature found for NMR analysis in fruits. For the postharvest quality of peaches and plums, 19 compounds have already been reported, and for tomatoes, 21 compounds [22]. For banana and jujube quality, the number of compounds identified were 11 and 22, respectively [6,23]. 

The characteristic polyphenol region containing numerous signals is also exhibited, which may be associated to the presence of proanthocyanidins, such as ellagitannins. These characteristic polyphenol signals have also been reported for plums and peaches [22]. The compound identification was possible due to the comparison of deconvoluted signals of ^1^H NMR spectrum of a sample with database, and confirmation with the interpretation of ^1^H-^1^H COSY NMR, ^1^H-^13^C HSQC NMR spectrum, and ^1^H JRES NMR spectrum as showed in Figure S1 (Supplementary Materials).

The NMR analysis in the present study was possible once careful sample preparation with pH control of the extracts and mathematical alignment of peaks was employed to prevent some compounds which are susceptible to interactions from affecting their chemical shifts and to keep the reproducibility. Additionally, the resolution was guaranteed by the carefully adjusted parameters such as calibrated pulse at 90° and the respective power to suppress water residual, correspond receiver gain (intensity), and the number of points.

For quantification, the spectra showed the metabolite amounts after the signal integration and the software table filled using the *Eretic2* method with metabolites and sample information (Figure S1, Supplementary Materials). The amounts of metabolites found will be discussed in the next section. When classified by accession (or plant), the spectra showed few changes between themselves (Figure S2, Supplementary Materials), also better explored in the next section.

### 3.3. Metabolite Amounts

Under appropriate quantitative NMR (qNMR) conditions, NMR spectra can provide direct quantitative information since the signal intensity of each resonance in the NMR spectrum is directly proportional to the number of equivalent nuclei responsible for that signal, or in other words, is directly proportional to the molar amount of the detected isotope [24,25]. Thus, with NMR it is possible to determine absolute concentrations, relying on the use of one internal, external, or electronic standard [25,26,27]. Using the one-dimensional (1D) ^1^H NMR spectroscopy as the conventional way to perform quantitative NMR analysis [28], we used the external 2 mM sucrose. Both sample and standard spectrum were measured under a set of appropriate conditions to obtain accurate results in quantitative analysis. As quantitative conditions depend on the targeted accuracy (trueness and precision), for a maximum error of 1%, a relaxation delay (delay before the excitation) was included that was equal to at least 5 times the longitudinal relaxation time (T1) for a 90° pulse, an acquisition time longer than 3 times the transverse relaxation time (T2), and a sufficient signal to noise (at least 50:1) [24,27]. Careful processing of NMR spectra was also required to extract accurate peak areas.

Sucrose, citric acid, and glutathione represented the main cambuci pulp constituents, comprising 61% of the total amount of the polar metabolites in this fruit (Table 2, Table 3 and Table 4).

Total sugar content (sucrose, glucose and fructose) represented 40% of all pulp metabolites. A balance between sugars is characteristic of each fruit species and decisively affects the quality perception experienced by consumers. Sucrose was the predominant sugar, although accession comparisons indicate a sucrose content variation of up to 3.9-fold (Table 2). Sucrose, fructose and glucose determine fruit sweetness, where fructose is perceived as sweeter, followed by sucrose and glucose [29]. In ‘Puket’ pineapples, the total sugar contents reach 13.2 g 100 g fw, with the sucrose amount being four times higher than that of glucose and fructose [30]. Strawberries containing 0.2–2.2 g 100 g^−1^ of sucrose, 1.7–2.5 g 100 g^−1^ of glucose and 1.6–2.9 g 100 g^−1^ of fructose [31] indicate that sweetness varies among cultivars. 

Glucose-6-phosphate, the precursor of most glucose-implicated pathways, was present in cambuci from 8.7 mg 100 g^−1^ to 51.2 mg 100 g^−1^ (Table 2). The operation of this pathway during the postharvest stage of the fruit is involved with the available energy [32,33,34]. For the cambucis evaluated, the quantification of glucose-6-phosphate was not directly related to the measured levels for the most common sugars in fleshy fruit: sucrose, fructose and glucose. Concerning related compounds, adenosine ribonucleoside and the sugar alcohol inositol were detected in lower amounts. These compounds have been reported in quality NMR analyses in minor amounts for several fruits, such as melons and peaches [35,36]. 

Ethanol content ranged from 3.5 mg 100 g^−1^ to 11.3 mg 100 g^−1^, indicating ongoing fermentation processes (Table 2). The presence of this compound was expected, since cambuci is a highly perishable fruit with a short postharvest life, indicating fermentation even before being detached from the plant. Ethanol results from the reduction of acetaldehyde (glycolysis result) during the anaerobic respiration process, responsible for the emission of volatile compounds described as strongly alcoholic, perceived in overripe fruits. High ethanol levels in newly harvested cambucis suggest that the control of this compound may be essential for the successful postharvest conservation of this fruit. Currently, cambucis are mostly consumed frozen. However, using conservation techniques that enable *in natura* consumption are promising, such as refrigeration and controlled and modified atmospheres.

Citric acid and shikimic acid were the most important organic acids in cambuci, representing 32% of all metabolites (Table 3). The citric acid content of cambucis is comparable to several citrus fruits, such as orange (560–980 mg 100 g^−1^) and grapefruit (1190–2100 mg 100 g^−1^), and is significantly higher than in apples (30–50 mg 100 g^−1^) [37]. Typical shikimic acid values in fruits may range from 0.3 mg 100 g^−1^ in apples and 84.4 mg 100 g^−1^ in jostaberries [37]. Nevertheless, reported cambuci values were higher than in these fruits, evidencing their peculiar composition. Furthermore, quinic acid is usually more abundant in fruits than shikimic acid, although this has not been confirmed for cambuci [37]. Accession 9 contained higher malic acid rates, while accession 6 contained the lowest amount among all evaluated accessions. Malic acid, which is derived from succinic acid, leads to a tart flavor. Quinic acid exhibited the lowest coefficient of variation (15%) in all cambuci accessions. Kiwi is considered a quinic acid-rich fruit, with reference values ranging from 400 to 1300 mg 100 g^−1^. The richest quinic acid cambuci accession contained 466.9 mg 100 g^−1^ (accession 4) (Table 3). Quinic, shikimic and gallic acid are known as polyphenol precursors. The mean amounts observed for polyphenols, quinic acid, shikimic acid, and gallic acid were 412.1 mg 100 g^−1^, 373.3 mg 100 g^−1^, 584.5 mg 100 g^−1^, and 4.1 mg 100 g^−1^, respectively (Table 3 and Table 5). Quinic acid together with gallic acid is involved in the early stage of tannin biosynthesis, which is associated with the typical astringent taste of cambucis [38]. Tannins are polyphenols belonging to the class of proanthocyanidins that occur in fruits and vegetables. The biosynthesis of tannins results in ellagitannin production, one of the major compounds of the phenolic class for this fruit, considered beneficial for human health, with a protective action against cardiovascular diseases and obesity [2].

Accession 6 and 8 contained the highest amounts of polyphenols, while accession 3 contained the lowest (Table 3). A paper assessing phenolic cambuci compounds indicated a potentially beneficial action concerning glucose intolerance attenuation and adipose tissue inflammation induced by a high-fat, high-sucrose diet [2]. Another study demonstrated polyphenols present in cambuci as important therapeutic actions that improve complications associated with obesity [39]. In fact, fruits from the botanical family Myrtaceae represent a rich source of secondary metabolites, which in nature are intrinsically involved in plant defense, especially biologically active polyphenols [40,41]. Alongside the shikimic acid contents reported herein, these findings point to a balance between polyphenols and shikimic acid (SHA) synthesis, revealing that this organic acid is probably directly involved in polyphenol synthesis. The SHA pathway in plants is responsible for generating numerous secondary metabolites [42]. It plays an important role as an intermediary for the biosynthesis of aromatic amino acids (tryptophan, tyrosine, and phenylalanine) and has extensive biotechnological applications, with analgesic, antioxidant, anticoagulant, anti-inflammatory, antithrombotic, neuroprotective effects and as material for the synthesis of antivirals [43,44,45,46,47]. 

Ascorbic acid (vitamin C), a natural antioxidant that prevents the action of free radicals, is an important organic acid in cambuci, averaging 47 mg 100 g^−1^, ranging from 26 mg 100 g^−1^ to 103 mg 100 g^−1^ (Table 3). These values are in agreement with previously reported data, where cambuci ascorbic acid contents ranging from 31.12 to 139.38 mg 100 g^1^ were described [3]. Therefore, cambuci is an excellent ascorbic acid source when compared to sweet orange (18.9–22.2 mg 100 g^−1^), mandarins (16.2–31.6 mg 100 g^−1^) and lemons (39.6–42.3 mg 100 g^−1^) [48]. The recommended daily dose (RDA) of vitamin C is of 75 (women) −90 (men) mg day^−1^, sufficient to meet cellular needs and reduce human health risks, such as cardiovascular and neurodegenerative diseases, cancer and stroke [49]. This finding could be explored in the food, cosmetics, and pharmaceutical industry as foods with increased vitamin C, cosmetics rich in natural vitamin C and complex vitamins including vitamin C could have a potential international market. 

Distinguishing sugar, organic acid and phenolic compound levels can contribute to the quality differentiation of fruits produced from different plants and aid in future efforts to breed cambucis. This type of differentiation has been previously reported for other cultivars, such as apples [50]. 

Amino acids’ relevance in plants arises from their role as central regulators of plant growth and responses to environmental signals, along with being agents that influence human food nutritional quality [51]. Eleven amino acids and related compounds were detected in cambuci (Table 4), where glutathione was the most noteworthy, representing 10% of all metabolites. Glutathione is a water-soluble antioxidant molecule consisting of three amino acids, glutamic acid, cysteine and glycine (a tripeptide). Glutamic acid amounts ranked second, always at least 2.5-fold lower than glutathione. Glutathione and glutamic acid were highly and positively correlated, with an index of 0.96. Glutamine was also positively correlated with glutamic acid, with an index of 0.94. Glutamic acid is the major amino acid of ripe fruits [52]. Glutathione, glutamic acid and glutamine displayed the lowest coefficients of variation among all detected amino acids.

Other amino acids and related compounds exhibiting a high positive correlation (>0.9) comprised gamma-aminobutyric acid (GABA) vs. isoleucine, GABA vs. valine, GABA vs. leucine, GABA vs. asparagine, isoleucine vs. valine, isoleucine versus leucine, isoleucine vs. asparagine, valine vs. leucine, and valine vs. asparagine. Amino acids in fruits are not only linked to protein constitution, but also contribute to organoleptic fruit qualities. For example, in tomatoes, the taste described as “umami” is directly associated to glutamic acid levels [53]. Amino acids are also the precursors of several volatile organic compounds, such as 2- and 3-methyl butanal, 2- and 3-methyl butanol, phenylacetaldehyde, 2-phenylethanol and methyl salicylate. [5].

The sugar and organic acid ratio (S/OA) ranged between 0.5 and 2.1, indicating both sweet and acidic fruits. The S/OA ratio was also correlated to the SSC/TA ratio, in which 0.5 (S/OA) corresponded to 3 (SSC/TA) and 2.1 (S/OA) to 8 (SSC/TA) (Table 5). The use of the S/OA ratio to replace the SSC/TA ratio is here presented as a new method to measure the quality attribute. Thus, NMR quantification of sugars and organic acids can be used to establish or replace the optimal values of the SSC/TA ratio of an unknown fruit such as cambuci, predicting the sweetness of the fruit, and guiding genetic breeding programs of the fruit. 

Furthermore, a lower sugar content was detected in accession 4, and lower organic acid and polyphenols, in accession 3. Accessions 3, 4, and 6 were obtained from the same city, under similar environmental conditions, which emphasizes possible genetic differences among producing plants.

**Figure 2:**
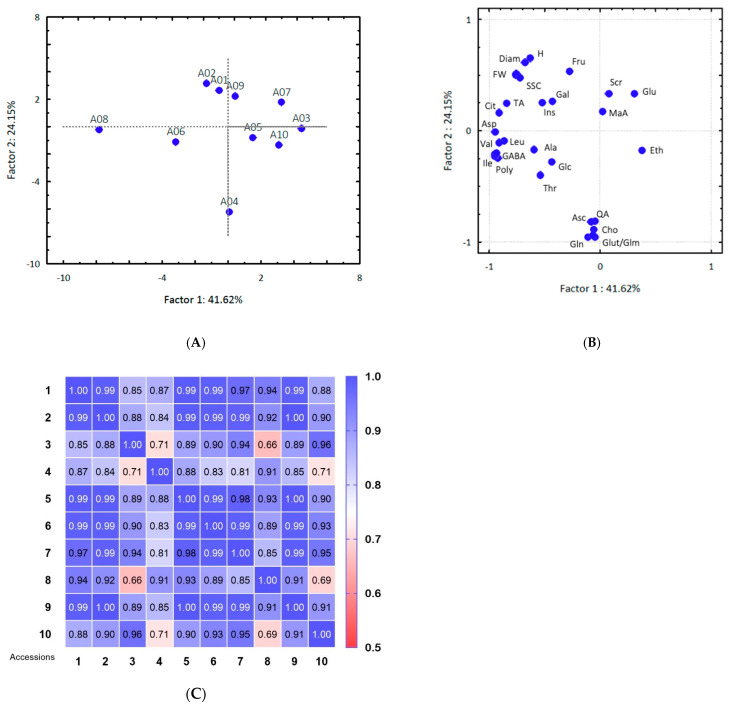
Principal component analysis showed scores (**A**) and loadings (**B**) and correlations (significant at *p* < 0.05) (**C**) including the parameters analyzed in cambuci pulp analytical and NMR analyses of the ten accessions named 1–10 (A01–A10). SSC: total soluble solids; TA: titratable acidity.; FW: fresh mass; H: height; Diam: diameter; Scr: sucrose, Glu: glucose, Fru: fructose, Glc: glucose-6-phosphate, Ado: adenosine, Ins: inositol, Eth: ethanol; Cit: citric acid, SHA: shikimic acid, QA: quinic acid, Asc: ascorbic acid, MaA: malic acid, Gal: gallic acid, Suc: succinic acid, Poly: polyphenols; Glut: glutathione, Gln: glutamine, Thr: threonine, GABA: gamma-aminobutyric acid, Ile: isoleucine, Val: valine, Ala: alanine, Leu: leucine, Cho: choline, Glm: glutamic acid, Asp: aspartic acid.

Accessions 3 and 4 stood out for their visible separation tendency while accessions 01 and 02, from Paraibuna-SP, showed high similarity (Figure 2A). Nevertheless, similarities and differences in fruit pulp profiles were observed between fruits harvested in the same municipality and between municipalities for the remaining accessions. Ethanol was relevant to distinguish accessions 3 from 4 (Figure 2B), gallic acid to distinguish accession 6 from 3 and 4. 

Therefore, the fruits could not be clearly separated by their municipality of origin. A range of 66% to 100% similarity in fruit pulp constituents was found by correlating parameters reported in this study (Figure 2C).

### 3.4. Metabolic Pathway of Common Metabolites

**Figure 3:**
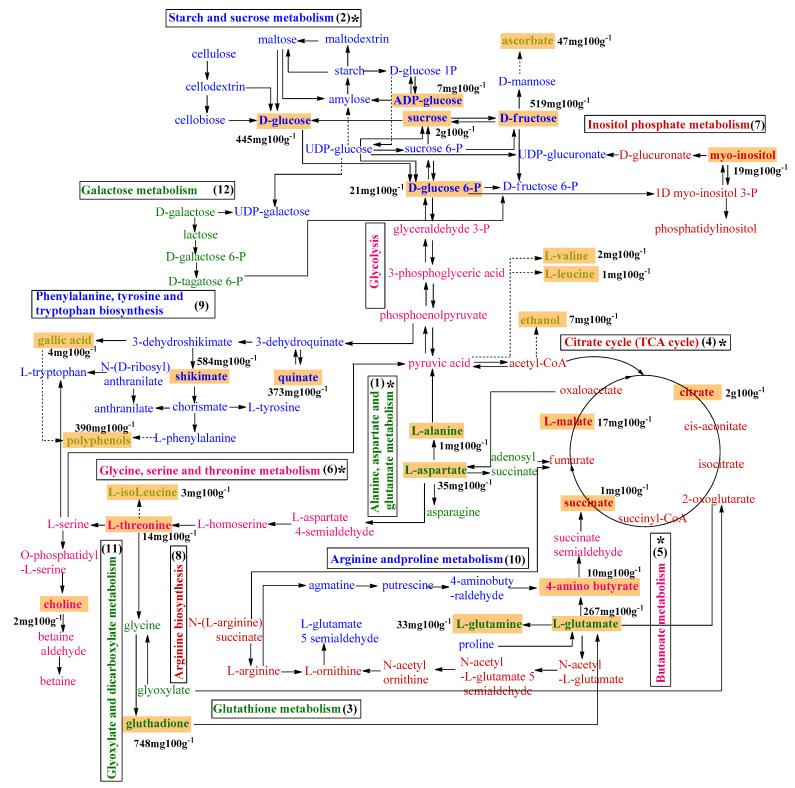
Pictorial representation of general metabolic cambuci pathways based on a pathway analysis. Cambuci metabolites are highlighted in an orange background with their average amounts, and their associated metabolisms are numbered by their impact factor order. The top five relevant pathways according to the –log (p) impact factors are marked with *. ADP: adenosine diphosphate, UDP: uridine diphosphate, GDP guanine diphosphate.

The metabolic pathway analysis indicated a total of 36 pathways (Table 6). The top 12 metabolic pathway of common cambuci metabolites described according to impact factor are displayed in Figure 3. 

When assessing the top 12 relevant pathways, only seven exhibited a pathway impact value higher than 0.1, as follows: (1) alanine, aspartate and glutamate metabolism, (2) starch and sucrose metabolism, (3) glutathione metabolism, (4) citrate cycle (TCA cycle), (5) butanoate metabolism, (6) glycine, serine and threonine metabolism, and (7) inositol phosphate metabolism, with impact values of 0.77, 0.53, 0.40, 0.16, 0.14, 0.12, and 0.10, respectively. Based on both their *p* values and impact values, this relevance can be further restricted to five metabolisms, namely (1), (2), (4), (5), and (6), with significant *p* values <0.05 (−log *p* > 3.15).

The alanine, aspartate and glutamate metabolism is the most relevant for cambucis, associated to their fundamental involvement as a central molecule of the amino acid metabolism in higher plants [54] (Table 6, Figure 3). Amino acids are involved in reactions in the primary and secondary metabolism of plants including those processes linked to ripening that directly deal with fruit quality once it has been harvested [55]. Amino acids are essential agents in physiological and biochemical reactions during maturation and senescence, having functions in the carbon and nitrogen cycle and playing vital roles during signaling processes in plant stress response [55]. Regarding fruit metabolism, it was described in figs under refrigeration that the behavior of glutamic acid and aspartic acid levels presents a positive correlation with increased soluble solids, glucose, fructose and the loss of fresh weight [56]. This relation was only observed with aspartic acid in cambucis, where accession 6 that presented the greatest amount of soluble solids, sucrose, glucose and fructose also had aspartic acid amounts higher than the mean (Table 4).

Sucrose, d-glucose and d-fructose are the main starch and sucrose metabolism components (Table 6, Figure 2). The balance between sugars is dependent on fruit genotypes, as noted for pomegranates, where variability in eight detected sugars was significantly high in different accessions [57]. This difference relates closely to the activity of the enzymes involved in the production of sugars, and therefore the sugar content of the fruit alters in line with the activity level of each one. The evaluated cambucis are fruits from wild accessions, which did not go through breeding, and therefore the sugar profile is related to what is found directly in nature. In this way, their sugars play roles essentially related to the survival of the species as sensing metabolism, fruit development, facilitating the formation of the cell wall, cell expansion, nourishes respiration and acid metabolism [58,59]. 

The third relevant cambuci metabolism was the TCA cycle, with malic acid, citric acid and succinic acid as the most noteworthy acids (Table 6, Figure 3). During fruit ripening, sugars may no longer be available for respiration, causing a change from sugars to organic acids (in particular citric acid) as a respiratory substrate [60]. In this phase, increased respiration may stimulate the conversion of citric acid into malate, in order to maintain the set of intermediate products in the TCA cycle constant [60]. All metabolic pathways linked to the TCA cycle depend on the transient formation of succinic acid, including the carbon metabolism and lipid, amino acid and GABA syntheses [61]. 

The fourth most representative pathway (butanoate metabolism) in cambuci is related to γ-aminobutyric acid (GABA, or 4-aminobutyrate), also derived from glutamic acid (Table 6, Figure 3). Glutamic acid is decarboxylated into GABA by the action of glutamic acid decarboxylate (GAD) in cytosol [54]. GABA plays a dual role as a signaling molecule and as a metabolite. Among other possibilities, stresses such as cold, heat, salt and soft or transient environmental factors such as touch, wind or rain, inherent to cambuci production and postharvest stages, are known to increase cellular Ca^2+^ levels [62]. In addition, the versatility of GABA in dealing with biotic stress factors shapes plant immune responses against pathogens by modulating the balance of reactive oxygen species in plant tissues [63]. 

Some amino acids are synthesized as a consequence of the association between GABA production and the C and N metabolism, both under both normal and stress conditions [64,65], such as threonine. Threonine is part of the glycine, serine and threonine metabolism, the fifth metabolic pathway recognized as important in cambucis. In tomatoes, a significant increase in threonine levels at the stage classified as mature green has been observed, which remained high until the end of this fruit’s postharvest life [66]. In kiwis, an increase in threonine in the fruit pulp was observed up to the stage classified as eating-ripe (soft), decaying from the stage classified as overripe [67]. Another nitrogen compound involved in this pathway is choline, an amino acid-like nutrient already described as one of the main metabolites responsible for the senescence in bananas [6].

## 4. Conclusions

This work reports, for the first time in the literature, the metabolite profile of cambucis measured by the NMR technique, focusing mostly on sugars, acids, amino acids and total polyphenols. This is also the first detailed description of amino acid composition in cambuci, adding that research addressing its sugars, acids and polyphenols are also scarce.

Sucrose is the predominant sugar, while glucose and fructose rank second, depending on the cambuci accession. Citric acid is the most common acid in cambuci, followed by shikimic and quinic acids in widely variable amounts. Cambuci contain ascorbic acid at levels comparable to citrus fruits, which make this fruit a rich source of this compound. Glutathione and glutamic acid are noteworthy among amino acids and their related compounds. According to the diverse metabolic profile described herein for cambuci, different sensorial experiences can be achieved by consuming this fruit. The quality richness described in the present study for the ten evaluated cambuci accessions offer valuable insights into future breeding programs and possible domestication actions for this fruit, leading to the possibilities of products of food, cosmetics and pharmaceutical industries.

## Figures and Tables

**Table 1:** Physicochemical cambuci quality parameters.

Accession	Fresh mass		Height		Diameter		SSC		TA		Ratio	
1	78.3	bc	42.4	cd	60.7	cd	11.0	cd	3.0	c	3.7	a
2	88.3	c	45.4	d	65.4	d	11.6	c	2.4	bc	4.8	ab
3	36.3	a	34.9	a	48.2	a	8.7	a	1.1	a	8.1	c
4	41.7	a	35.7	ab	47.6	a	7.8	a	2.2	bc	3.5	a
5	44.5	a	37.1	abc	51.8	ab	9.0	ab	2.4	bc	3.8	a
6	57.6	ab	42.2	cd	52.0	ab	14.0	f	2.6	c	5.7	abc
7	44.4	a	38.6	abc	53.0	ab	10.7	bcd	2.4	bc	4.4	ab
8	86.0	c	41.9	cde	66.4	d	13.4	ef	4.3	d	3.2	a
9	53.7	a	42.2	cd	56.4	bc	12.2	de	3.2	c	3.9	a
10	44.9	a	36.2	abc	52.1	ab	9.3	abc	1.5	ab	6.6	bc
Mean	57.0		39.6		55.1		10.8		2.5		4.8	
CV (%)	26.2		10.0		8.5		8.6		14.7		18.0	

Values comprise the mean of three repetitions (where each repetition contained ten fruits) for fresh mass, height, diameter and three repetitions for SSC, TA, ratio (where each repetition contained three fruits). Different lowercase letters within a column indicate a significant difference at *p* < 0.05. CV: coefficient of variation. SSC: total soluble solids; TA: titratable acidity. Fresh mass: g; height and diameter: mm; SSC: °Brix; TA: % citric acid eq.

**Table 2:** Means (fruits quintuplicate for each accession) of the sugars and related compounds determined by NMR and expressed in mg 100 g^−1^.

Accession	SCR		GLU		FRU		GLC		ADO		INS		ETH	
1	1553.0	ab	460.2	abc	426.5	abc	17.1	a	6.4	abc	11.6	a	6.8	ab
2	2080.1	bcd	444.7	abc	611.9	bc	24.0	a	6.0	ab	40.2	a	6.5	ab
3	1756.8	abc	571.7	bc	624.0	bc	26.2	a	2.2	a	18.2	a	11.3	b
4	784.0	A	220.9	a	284.4	a	23.1	a	6.1	ab	22.6	a	5.5	ab
5	1908.1	bc	481.8	abc	481.0	abc	11.5	a	4.2	ab	6.6	a	8.8	ab
6	3098.2	d	693.5	c	695.5	c	51.2	b	11.1	bc	3.2	a	9.8	ab
7	2073.1	bcd	416.8	ab	489.3	abc	9.9	a	2.5	ab	19.7	a	5.5	ab
8	1482.6	ab	235.4	a	571.7	abc	25.2	a	15.1	c	57.1	a	4.9	ab
9	2108.2	bcd	470.7	abc	656.8	c	8.7	a	9.0	abc	44.5	a	3.5	a
10	2617.0	cd	451.8	abc	348.9	ab	17.6	a	3.4	ab	13.0	a	9.2	ab
Mean	1946.1		444.7		519.0		21.5		6.6		23.7		7.2	
CV (%)	25.4		27.7		26.0		51.2		63.6		135.9		48.6	

Different lowercase letters within a column indicate a significant difference at *p* < 0.05. CV: coefficient of variation. Scr: sucrose, Glu: glucose, Fru: fructose, Glc: glucose-6-phosphate, Ado: adenosine, Eth: ethanol, Ins: inositol.

**Table 3:** Means (fruits quintuplicate for each accession) of organic acids and related compounds determined by NMR and expressed in mg 100 g^−1^.

Accession	CIT		SHA		QA		ASC		MAA		GAL		SUC		POLY	
1	1614.5	bcd	549.7	a	265.7	a	41.7	a	15.1	ab	5.3	abcd	0.6	a	482.1	abc
2	1966.2	cd	461.5	a	325.2	ab	25.9	a	18.5	ab	9.5	d	0.6	a	374.0	abc
3	617.0	a	370.7	a	346.4	abc	40.8	a	12.3	ab	2.0	abc	0.2	a	8.7	a
4	1400.3	abc	521.3	a	466.9	d	103.1	b	18.5	ab	5.9	bcd	0.7	ab	509.7	abc
5	1843.3	bcd	662.0	ab	383.8	abc	38.4	a	13.7	ab	2.0	abc	0.6	a	340.7	abc
6	2385.2	de	928.3	bc	464.0	cd	56.7	a	6.8	a	1.5	ab	0.8	ab	750.8	bc
7	1484.9	abcd	464.7	a	333.4	ab	29.0	a	14.4	ab	1.8	abc	0.2	a	143.9	a
8	2934.4	e	1012.8	c	355.6	abcd	40.5	a	18.5	ab	5.5	abcd	2.5	b	836.8	c
9	1969.0	cd	559.3	a	366.5	abcd	53.9	a	32.3	bc	6.2	cd	0.5	a	281.2	ab
10	934.0	ab	315.0	a	425.5	bcd	41.3	a	21.1	bc	1.1	a	0.3	a	314.6	ab
Mean	1714.9		584.5		373.3		47.1		17.1		4.1		0.7		412.1	
CV (%)	26.0		27.9		15.0		42.9		87.4		51.7		121.6		57.3	

Different lowercase letters within a column indicate a significant difference at *p* < 0.05. CV: coefficient of variation. Cit: citric acid, SHA: shikimic acid, QA: quinic acid, Asc: ascorbic acid, MaA: malic acid, Gal: gallic acid, Suc: succinic acid, Poly: polyphenols.

**Table 4:** Means (fruits quintuplicate for each accession) of amino acids and related compounds determined by NMR and expressed in mg 100 g^−1^.

Accession	GLUT		GLN		THR		GABA		ILE		VAL		ALA		LEU		CHO		GLM		ASP	
1	509.1	a	24.5	A	13.4	a	12.6	ab	4.1	ab	2.5	ab	0.6	a	1.5	ab	1.2	a	179.9	a	34.2	ab
2	578.6	ab	28.8	A	14.9	ab	10.1	ab	3.5	ab	2.2	ab	2.5	a	1.3	ab	1.4	a	193.6	a	34.0	ab
3	711.3	ab	33.0	A	9.0	a	5.1	a	1.3	a	0.7	a	2.2	a	0.3	a	1.6	ab	265.1	a	13.4	a
4	1122.0	c	52.0	B	17.1	ab	12.6	ab	4.2	ab	2.5	ab	2.6	a	1.5	ab	2.7	b	429.9	b	35.9	ab
5	738.6	ab	32.3	A	11.9	a	8.9	ab	3.1	ab	1.8	ab	0.6	a	1.0	ab	2.2	ab	274.1	a	37.7	ab
6	847.1	bc	34.3	A	26.7	b	17.8	ab	5.7	ab	3.5	ab	0.5	a	2.2	ab	1.5	a	275.9	a	59.1	bc
7	655.2	ab	26.7	A	11.2	a	2.0	a	0.8	a	0.3	a	1.0	a	0.1	a	1.5	a	204.8	a	17.3	a
8	764.2	ab	34.9	A	16.8	ab	25.9	b	7.8	b	4.7	b	4.8	a	2.8	b	2.0	ab	285.6	a	80.0	c
9	704.4	ab	27.2	A	5.7	a	6.7	a	2.2	ab	1.3	ab	0.5	a	0.7	ab	1.4	a	272.2	a	20.0	a
10	849.9	bc	34.2	A	15.0	ab	7.5	a	2.4	ab	1.4	ab	0.0	a	0.8	ab	1.9	ab	289.2	a	25.6	a
Mean	748.0		32.8		14.2		10.9		3.5		2.1		1.4		1.2		1.7		267.0		35.7	
CV (%)	17.4		19.7		39.6		74.5		73.7		81.5		212.2		86.1		31.1		19.5		43.5	

Different lowercase letters within a column indicate a significant difference at *p* < 0.05. CV: coefficient of variation Glut: glutathione, Gln: glutamine, Thr: threonine, GABA: gamma-aminobutyric acid, Ile: isoleucine, Val: valine, Ala: alanine, Leu: leucine, Cho: choline, Glm: glutamic acid, Asp: aspartic acid.

**Table 5:** Means of metabolite groups, sugars, organic acids, amino acids, polyphenols and sugar organic acid ratio (S/OA) determined by NMR and expressed in mg 100 g^−1^.

Accession	Sugar		Rel. Sugar		Org Acids		Am Acids		Poly		Sum		S/OA
1	2439.6	ab	41.9	a	2492.7	abc	783.7	a	482.1	abc	6240.0	a	1.0
2	3136.8	bc	76.7	a	2807.3	bcd	870.9	abc	374.0	abc	7265.6	ab	1.1
3	2952.5	b	58.0	a	1389.2	a	1043.2	abc	86.9	a	5529.7	a	2.1
4	1289.2	ab	57.4	a	2516.7	abc	1683.1	d	509.7	abc	6056.1	a	0.5
5	2870.8	b	31.1	a	2943.9	cd	1112.1	abc	340.7	abc	7298.6	ab	1.0
6	4487.2	c	75.4	a	3868.8	de	1273.3	c	750.8	bc	10455.5	c	1.2
7	2979.3	b	37.7	a	2328.3	abc	921.1	abc	143.9	a	6410.3	ab	1.3
8	2289.6	ab	102.3	a	4369.9	e	1229.6	bc	836.8	c	8828.1	bc	0.5
9	3235.6	bc	65.8	a	2962.2	cd	1042.2	abc	281.2	ab	7587.1	ab	1.1
10	3417.8	bc	43.2	a	1738.3	ab	1227.6	bc	314.6	ab	6741.6	ab	2.0
Mean	2909.9		58.9		2741.7		1118.7		412.1		7241.2		1.2
CV (%)	23.8		58.2		19.8		15.8		57.3		15.9		44.9

Values comprise the means of five repetitions. Different lowercase letters within a column indicate a significant difference at *p* < 0.05. Sugars comprise the sum of Scr, Fru, Glu; related sugars comprise the sum of Glc, Ins, Ado, Eth; amino acids represent the sum of Thr, Ala, Glut, Gln, Val, Leu, Ile, GABA, Cho, Glm, Asp; organic acids comprise the sum of Asc, MaA, Cit, Suc, QA, SHA, Gal; polyphenols comprise the sum of the aromatic spectrum region. Legend. S/OA: sugar/organic acid ratio.

**Table 6:** Result from pathway analysis order by impact factor relevance.

	Pathway	Total	Expected	Hits	Raw p	-log(p)	Holm adjust	FDR	Impact
1	Alanine, aspartate and glutamate metabolism	22	0.37087	6	7.56 × 10^−7^	14.095	7.18 × 10^−5^	3.63 × 10^−5^	0.77338
2	Starch and sucrose metabolism	22	0.37087	3	0.0052788	5.244	0.4751	0.072396	0.52723
3	Glutathione metabolism	26	0.4383	2	0.069283	26.696	1	0.44341	0.40159
4	Citrate cycle (TCA cycle)	20	0.33715	2	0.043066	3.145	1	0.31803	0.15581
5	Butanoate metabolism	17	0.28658	3	0.0024648	60.057	0.22676	0.046798	0.13636
6	Glycine, serine and threonine metabolism	33	0.5563	3	0.016545	41.017	1	0.15883	0.1204
7	Inositol phosphate metabolism	28	0.47202	2	0.078956	25.389	1	0.47374	0.10251
8	Arginine biosynthesis	18	0.30344	3	0.0029249	58.345	0.26616	0.046798	0.08544
9	Phenylalanine, tyrosine and tryptophan biosynthesis	22	0.37087	1	0.3139	11.587	1	1	0.08008
10	Arginine and proline metabolism	34	0.57316	2	0.11026	22.049	1	0.58806	0.07781
11	Glyoxylate and dicarboxylate metabolism	29	0.48887	4	0.0011247	67.902	0.1046	0.026993	0.06012
12	Galactose metabolism	27	0.45516	3	0.0094826	46.583	0.84395	0.11379	0.04805
13	Sulfur metabolism	15	0.25287	1	0.22605	1.487	1	0.9435	0.03315
14	Phosphatidylinositol signaling system	26	0.4383	1	0.35971	10.224	1	1	0.03285
15	Glycerophospholipid metabolism	37	0.62374	1	0.47106	0.75277	1	1	0.03075
16	Purine metabolism	63	1.062	2	0.28755	12.464	1	1	0.00126
17	Aminoacyl-tRNA biosynthesis	46	0.77546	8	3.34 × 10^−7^	14.912	3.21 × 10^−5^	3.21 × 10^−5^	0
18	Valine, leucine and isoleucine biosynthesis	22	0.37087	4	0.00037527	78.879	0.035276	0.012009	0
19	Nitrogen metabolism	12	0.20229	2	0.016243	41.201	1	0.15883	0
20	Valine, leucine and isoleucine degradation	37	0.62374	3	0.022537	37.926	1	0.19669	0
21	Ascorbate and aldarate metabolism	18	0.30344	2	0.035399	33.411	1	0.28319	0
22	Carbon fixation in photosynthetic organisms	21	0.35401	2	0.047114	30.552	1	0.32307	0
23	Glucosinolate biosynthesis	65	10.958	3	0.092973	23.754	1	0.52502	0
24	Monobactam biosynthesis	8	0.13486	1	0.12745	2.06	1	0.64396	0
25	Lysine biosynthesis	9	0.15172	1	0.14224	19.502	1	0.68275	0
26	Selenocompound metabolism	13	0.21915	1	0.19903	16.143	1	0.86847	0
27	Nicotinate and nicotinamide metabolism	13	0.21915	1	0.19903	16.143	1	0.86847	0
28	beta-Alanine metabolism	18	0.30344	1	0.26494	13.282	1	1	0
29	Propanoate metabolism	20	0.33715	1	0.28983	12.385	1	1	0
30	Pantothenate and CoA biosynthesis	23	0.38773	1	0.32564	1.122	1	1	0
31	Glycolysis / Gluconeogenesis	26	0.4383	1	0.35971	10.224	1	1	0
32	Cyanoamino acid metabolism	29	0.48887	1	0.39213	0.93616	1	1	0
33	Pyrimidine metabolism	38	0.64059	1	0.48021	0.73354	1	1	0
34	Cysteine and methionine metabolism	46	0.77546	1	0.5481	0.6013	1	1	0
35	Porphyrin and chlorophyll metabolism	48	0.80917	1	0.56369	0.57325	1	1	0
36	Amino sugar and nucleotide sugar metabolism	50	0.84289	1	0.57876	0.54686	1	1	0

## Data Availability

Data available on request.

## Supplementary Materials

The supplementary materials available online.

## Sample Availability

Not available.
