# Supplementary material for: Classical Food Quality Attributes and the Metabolic Profile of Cambuci, a Native Brazilian Atlantic Rainforest Fruit
Source: Molecules. 2021 Jun 12;26(12):3613. doi: 10.3390/molecules26123613 (PMC8231640; doi:10.3390/molecules26123613)
Supplement: Supplementary file 1 [file molecules-26-03613-s001.zip › molecules-1233171-supplementary.pdf]

Supplementary material

Classical food quality attributes and the metabolic profile of cambuci, a native Brazilian Atlantic Rainforest fruit

Poliana Cristina Spricigo[1*], Banny Silva Barbosa Correia[2], Karla Rodrigues Borba[3], Isabela Barroso Taver[1], Guilherme de Oliveira Machado[2], Renan Ziemann Wilhelms[4], Luiz Henrique Keng Queiroz Junior[4], Angelo Pedro Jacomino[1*], Luiz Alberto Colnago[3].

[1] Luiz de Queiroz College of Agriculture, University of São Paulo. 11 Pádua Dias Ave. Zip Code 13418-900, Piracicaba, São Paulo, Brazil.

[2] Institute of Chemistry of Sao Carlos, University of São Paulo. 400 Trabalhador São Carlense Ave. Zip Code 13566-590, São Carlos, São Paulo, Brazil.

[3] Embrapa Instrumentation, 1452 XV de Novembro Street, Zip Code 13560-970, São Carlos, São Paulo, Brazil.

[4] Chemistry Institute, Federal University of Goiás. Esperança Ave, Zip Code 74690-900, Goiânia, Goiás, Brazil.

*Correspondence: polianaspricigo@usp.br; Tel.: +55 19 3429-4110

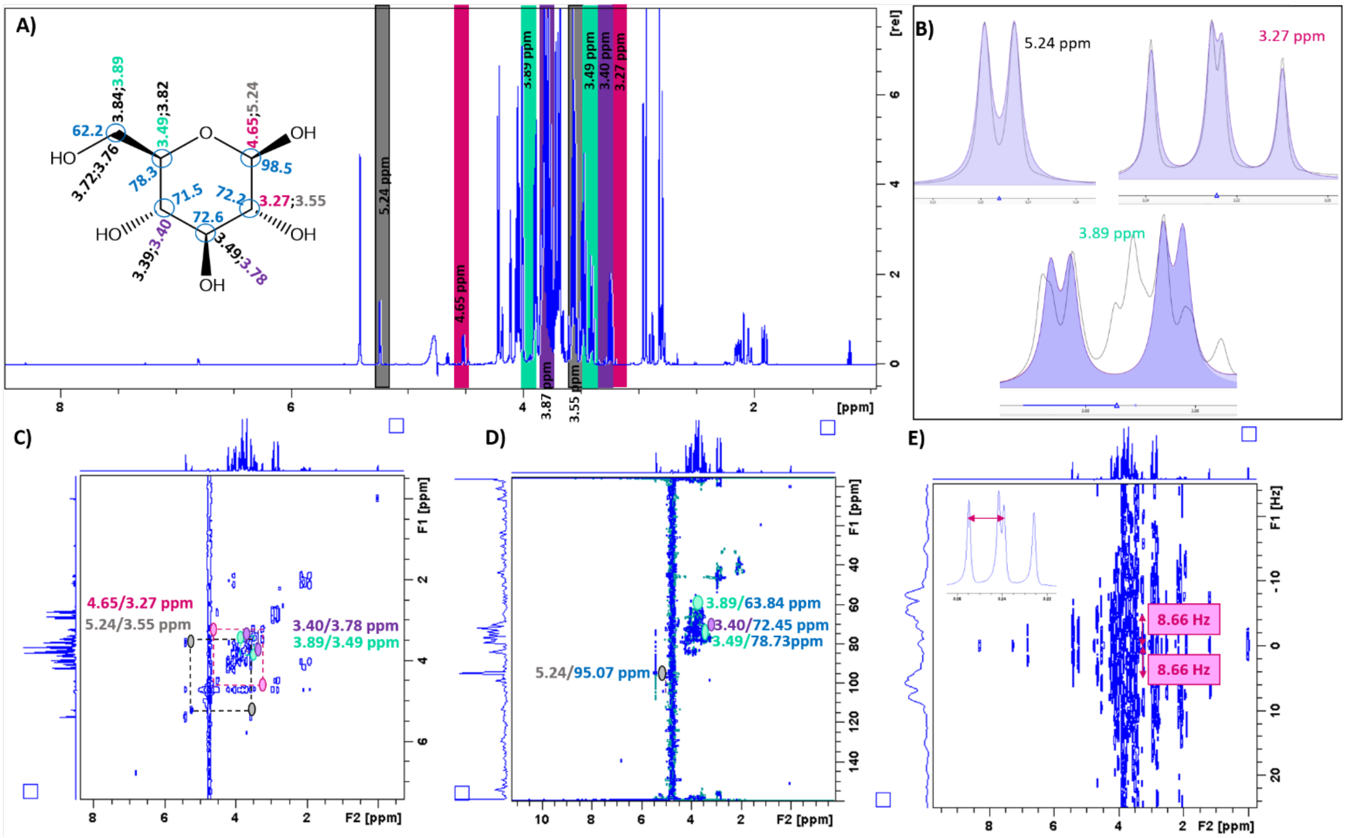

Figure S1. The typical process of identification of one compound (D-glucose) in the biological matrix (Cambuci pulp). A) ^{1}H NMR spectrum highlighting with colors the assignment according to the molecule, B) overlap of main glucose signals with signals in the sample showing deconvolution in blue and black the raw signal of the sample, C) ^{1}H, ^{1}H COSY NMR spectrum showing correlated signals between vicinal hydrogens, D) ^{1}H, ^{13}C HSQC NMR spectrum showing correlated signals between carbon and correspondent hydrogen, and E) ^{1}H JRES NMR spectrum showing J-coupling constant between protons of a specific signal.

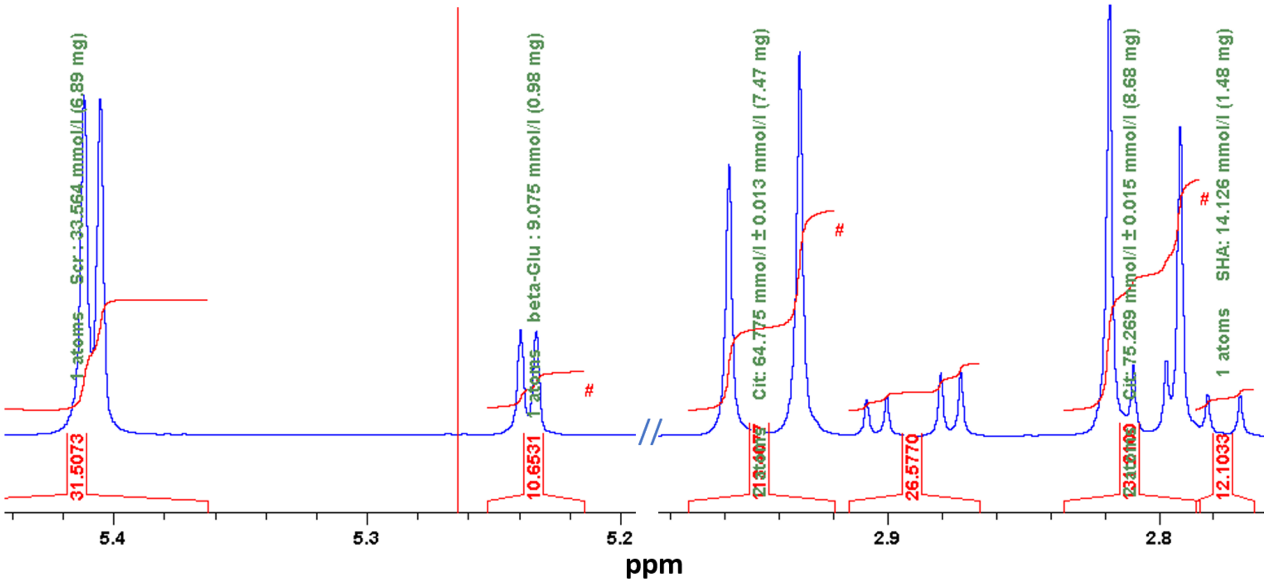

Figure S2. Quantitative ¹H NMR spectrum of the cambuci supernatant (accession 01) showing some signals with their respective assignment and amounts (Scr: Sucrose, Glu: D-Glucose, Cit: Citric acid, SHA: Shikimic acid).

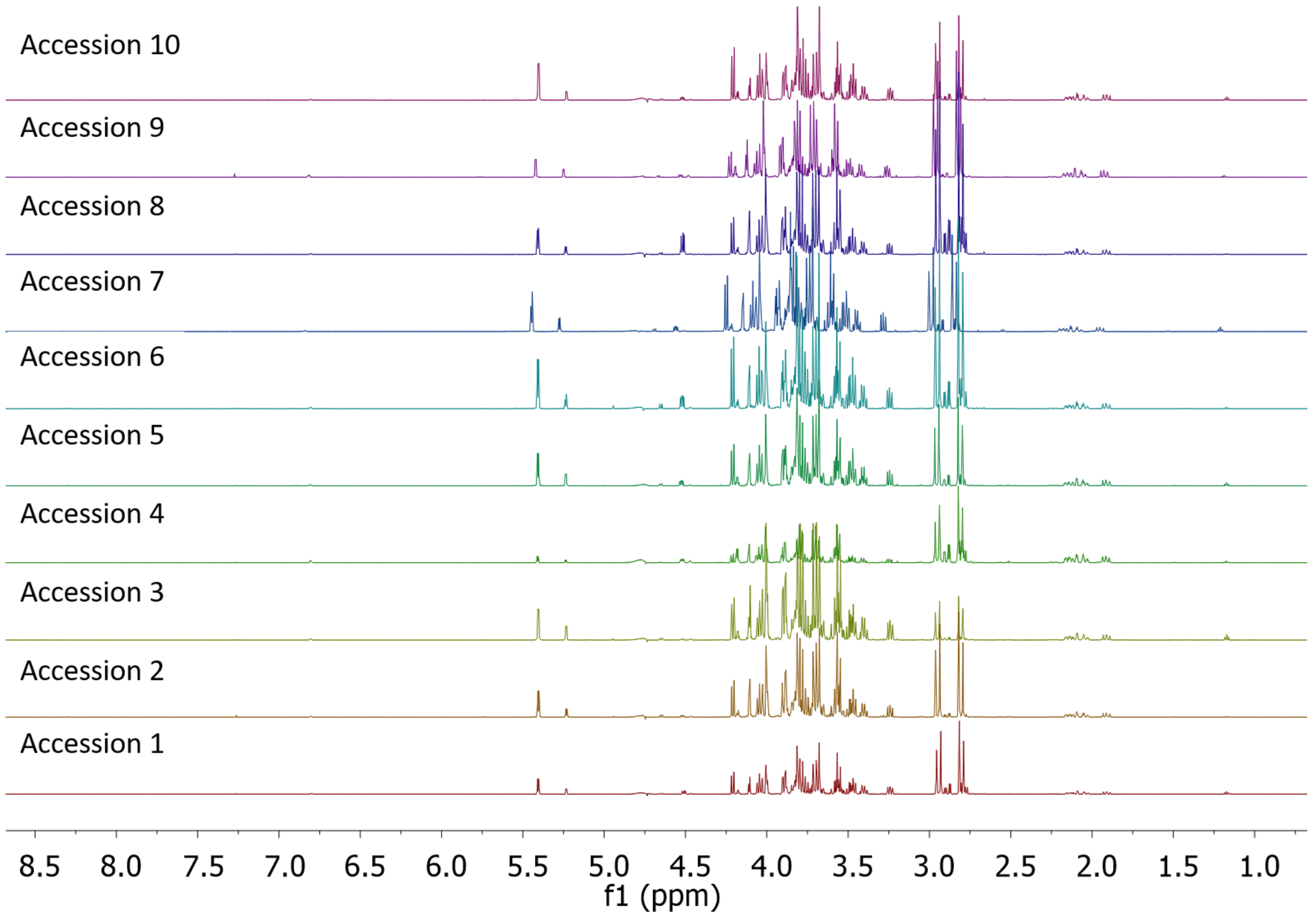

Figure S3. Typical ¹H NMR spectrum of supernatants from the fruit pulp of ten accessions analyzed in this study.
